# Understanding perinatal vulnerabilities: how Aboriginal women’s cultural strengths and resilience shapes their social and emotional wellbeing

**DOI:** 10.3389/fpubh.2025.1677055

**Published:** 2025-11-13

**Authors:** Patricia Ratajczak, Tracy Reibel, Ailsa Munns, Roz Walker, Rhonda Marriott

**Affiliations:** Ngangk Yira Institute, Murdoch University, Perth, WA, Australia

**Keywords:** Aboriginal women, perinatal screening, culturally safe care, digital innovation (DI), resilience in parents, cultural strengths, social and emotional wellbeing (SEWB)

## Abstract

**Background:**

During pregnancy, childbirth and postnatally, women are at their most vulnerable, requiring health and social care systems able to meet their needs. In the context of perinatal care, assessing Aboriginal women’s mental health requires consideration of their whole-of-life to establish their overall social and emotional wellbeing. This requires mechanisms which respect women’s cultural positioning and needs. In the Australian health care system, Aboriginal women’s mental health is routinely viewed through mainstream screening and assessment tools, such as the Edinburgh Postnatal Depression Scale which does not address cultural strengths or the protective nature of being connected to culture. In the face of significant structural inequities, including in perinatal care, Aboriginal women are frequently marginalized which contributes to their disengagement from services. Despite this, women’s resilience remains evident and understanding why may hold the key to better perinatal care planning. As such, the aim of this study was to explore Aboriginal women’s resilience, self-efficacy and empowerment during their perinatal experiences, assessing factors contributing to their cultural strengths when addressing perinatal mental health concerns.

**Methods:**

situated in a larger pilot implementation project, this qualitative study used an Aboriginal Participatory Action Research method and was undertaken on Whadjuk Country, Boorloo (Perth Western Australia). Aboriginal women (*n* = 8) were invited to participate in yarns with the study’s lead Aboriginal researcher. Data was inductively and deductively analyzed, with findings interpreted through a decolonizing framework which prioritized strengths and cultural ways of being.

**Results:**

Six themes were identified from analysis of the qualitative data: (1) strengthening identity-reconnecting to Culture; (2) connection to kinship/family sub-theme, strong partner support; (3) connection to country; (4) connection to culture; (5) resilience and self-efficacy; and (6) women’s experiences using the Baby Coming You Ready program’s digital platform. Themes 1–5 clearly demonstrated women’s strengths and resilience which were reported as a direct result of their culture and cultural connections; while theme 6 reported their positive experiences of using a strengths-based and culturally developed perinatal assessment platform.

**Conclusion:**

The results of this study confirm the positive benefits and value of co-designing tools for use in clinical settings which incorporate the cultural determinants of health and holistic perspectives of social and emotional wellbeing when screening Aboriginal women’s perinatal mental health.

## Background

During pregnancy, childbirth and postnatally, women are at their most vulnerable, requiring health and social care systems able to meet their needs. In the context of perinatal care, assessing Aboriginal women’s mental health requires consideration of their whole-of-life to establish their overall social and emotional wellbeing (SEWB). Mechanisms which respect women’s cultural positioning and strengths are needed. In the Australian health care system, Aboriginal women’s perinatal mental health is routinely viewed through mainstream screening and assessment tools, such as the commonly used Edinburgh Postnatal Depression Scale (EPDS). The EPDS has not been validated for use with Aboriginal women and does not reflect on cultural strengths and the protective nature of being connected to culture. Significant structural inequities, including in perinatal care, marginalize Aboriginal women, contributing to their disengagement from services. Despite this, women’s resilience remains evident and understanding why may hold the key to better perinatal care planning (1).

**Figure 1:**
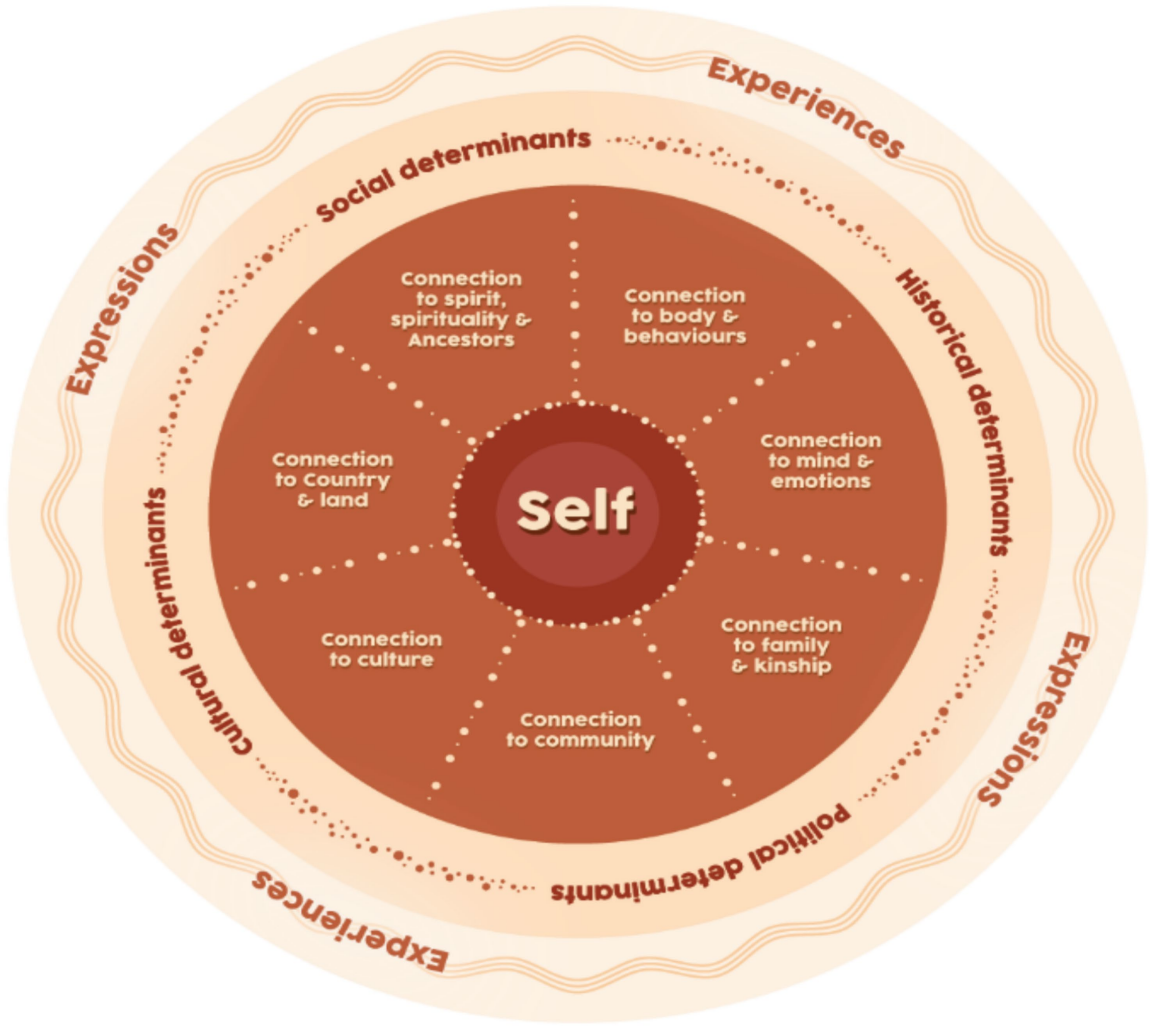
Social and emotional wellbeing model. Adapted with permission from “Social and Emotional Wellbeing from an Aboriginal and Torres Strait Islanders’ Perspective” by Gee et al. (2).

Aboriginal people hold a whole-of-life, relational worldview, described as social and emotional wellbeing (SEWB) and articulated through nine guiding principles (2) (see Figure 1). Dudgeon et al. (3) describe SEWB as cultural strengths realized through connection to Country, spirituality, ancestry, kinship, strong community and culture to mitigate psychological distress. The ability to effectively support Aboriginal women’s perinatal social and emotional wellbeing is therefore crucial in their attainment of immediate and longer term physical and psychosocial health and development (4). However, in seeking their best outcomes, Aboriginal women need to navigate the impacts of structural inequities and interpersonal, institutional and systemic racism on their mental health and wellbeing (5, 6).

SEWB is also strongly influenced by social and cultural determinants impacting on the health and wellbeing of individuals, families and communities; with the social determinants of health referring to the conditions in which people live and work influencing their control over self-determination, income and resources. As a consequence, if social determinants impacts are ignored, health inequities arise, causing notable differences in health status between population groups and countries (7). In contrast cultural determinants of health are strength-based elements based on Aboriginal knowledge and cultural identity, functioning as protective factors for individual and collective health and wellbeing (8).

Racism is a strong predictor of disempowerment and health and social inequities, perpetuating significant physical and psychosocial disparities (9). Over time, chronic stress diminishes SEWB, while adverse mental health and psychosocial outcomes are strongly linked to experiences of societal and organizational racism. The impacts of racism on individuals have been described as producing higher allostatic loads—referring to the cumulative physiological burden on the body from repeated stress—contributing to intergenerational epigenetic changes with risks for lifelong health trajectories (10, 11, 47). However, emerging evidence suggests that self-determination achieved by authentic Aboriginal leadership in the development of responsive strategies and culturally informed/co-designed approaches to health care, including in mental health, may combat stress produced by repeated exposure to racism (5). The inclusion of cultural determinants in clinical screens used to establish Aboriginal women’s perinatal mental health is more likely to support their positive mental health outcomes when health care pathways are combined with referrals to appropriate support services that help women access health care and social support which benefits them and their infants (12).

There is limited documented evidence on Aboriginal women’s perspectives of what constitutes positive mental health, or SEWB, and how this may be optimally assessed, particularly in relation to incorporating supporting cultural determinants of health. Additionally, both cultural and social determinants are broadly overlooked in clinical settings, while health system operational silos and poor interagency communication further disadvantage Aboriginal mothers (12). From the limited evidence it is clear that perinatal Aboriginal women cannot be appropriately supported unless there is a means of effectively engaging women in their own care. A means of encouraging their disclosure of emotional or social concerns to their health care provider and enabling shared decision making regarding the options available to address their self-identified concerns into care planning are needed to reverse inequitable perinatal outcomes.

### Aboriginal women’s strength and resilience

This study focused on how Aboriginal women’s strengths and resilience impact perinatal SEWB concerns. The research, a sub-study in a larger pilot implementation project [Baby Coming You Ready (BCYR)] investigated how strength-based approaches to assessing SEWB in the perinatal period empowered women to identify self-directed strategies to manage their concerns. The study outcomes are informative for health professionals caring for Aboriginal women, providing insight into how they might work in partnership with women to ensure culturally safe pathways to equitable maternal perinatal health care.

Current universal perinatal screening has a Western biomedical focus on risk, in contrast to strength-based approaches recognizing individuals’ self-efficacy and resilience acting as protective factors. The EPDS is the most prevalent screening tool for perinatal mental health in Australian maternity health settings (13). Known to have limitations, including not being validated for use with Aboriginal women (14, 15); studies have confirmed the EPDS does not identify Aboriginal women’s self-efficacy, strengths or resilience (16, 17). As a result, being exposed to the EPDS during perinatal care discourages Aboriginal women’s disclosure of concerns and worries leading to inaccurate clinical assessments about their mental health which results in poor follow-up (18, 19, 46). Further, considerations such as understanding why Aboriginal women do not trust mainstream health services (1, 12, 20), alongside their anxiety of being referred to social workers and/or child protection services, need to be considered. Also needed is for health professionals to develop their professional capabilities to work with Aboriginal women in culturally safe partnerships (19, 21).

The gap between Aboriginal and non-Aboriginal perinatal outcomes will not be reduced until approaches to routine screens incorporate understanding of protective factors, such as women’s connections to family, kinship, community support, culture and heritage, all of which have a vital role in the development of resilience (20, 48). While contemporary concepts of resilience are largely framed by Western understandings, a recent Australian scoping review (22) explored Aboriginal perspectives and culturally informed coping mechanisms related to resilience. To foster resilience, Usher and colleagues identified the need for strategies which strengthen individual connections to culture, including insights and culturally inclusive understandings of resilience from individual, social and cultural/community perspectives. It has been asserted that protective factors incorporated into holistic, culturally sensitive perinatal care approaches will strengthen the capacity of Aboriginal women to navigate their way through self-identified psychosocial and cultural wellbeing support (23). This being the case would likely contribute to reduced impacts from immediate and long-term inequities and marginalization faced by Aboriginal women and their families (24).

Poor health care interactions and outcomes have been noted where non-Aboriginal health professionals have difficulty empathizing with Aboriginal clients. For example, the racial empathy gap manifested through a lack of cultural sensitivity and emotional disengagement from healthcare workers toward clients (25) impacts the provision of culturally safe perinatal mental health care for Aboriginal women with a greater incidence of disparities in their journeys of care (26, 27). By contrast, engaging with Aboriginal women to co-design enhanced culturally safe, strength-based reflexive models of perinatal care ensures their cultural beliefs and practices are respected, enabling empowerment to express their needs and preferences (25, 28, 29). Six key dimensions have been identified to guide health professionals in establishing reflective, decolonizing strategies which meet the needs and aspirations of global populations, including Aboriginal peoples in Australia. These are: listening respectfully, building respectful relationships, using appropriate communication skills, reflecting critically on Australian political, social and historical contexts, applying a human rights-based approach and evaluating the processes and outcomes of service delivery (3, 30, 31).

The aim of this research was to explore Aboriginal women’s resilience, self-efficacy and empowerment during their perinatal experiences, assessing factors contributing to their cultural strengths when addressing perinatal mental health concerns. It was also anticipated the findings could influence health professionals and health organizations to more proactively consider the benefits of understanding cultural strengths when working with the women and their families.

### Research context

This study was undertaken by the Aboriginal lead researcher, with the support of two expert non-Aboriginal senior researchers in qualitative research in Aboriginal contexts. The study was situated within a larger evidenced-based Aboriginal-led culturally safe perinatal assessment pilot implementation study based on the *Baby Coming You Ready* (BCYR) digital platform. In partnership with perinatal health services, the co-designed BCYR digital platform was provided as an alternative to routine screens. Scheduled as a stand-alone appointment following closely after the initial antenatal intake visit, the BCYR digital platform guides Aboriginal mothers through self-reflective, strength-based assessments of social, emotional and cultural health determinants using a culturally safe system of online perinatal assessment rubrics embedded in the BCYR digital platform. Integral to the functioning of the assessment rubrics is the use of touch-screen visual prompts for mothers which guide yarning conversations, automatically populating a visual personalized story for mothers across a range of psychosocial and cultural issues. The yarning style of delivery promoted by BCYR culturally aligns with Aboriginal people’s routine use of storytelling in everyday conversations, while images reflecting Aboriginal people are used as prompts for women to select images which most closely reflect their current circumstances. Importantly, the woman holds the device on which the digital platform is operated, choosing images in response to the questions, posed either by an optional audio explanation or by the health professional. The purposeful approach ensures that women have control over what questions they want to respond to, and by making image selections most closely associated with their experience. The health professional sits alongside the woman, observing responses, clarifying if required and prompting with further questions as relevant. The initial part of BCYR focuses on the woman’s “life story”—for example, who she grew up with, her networks of support and connections to culture—advancing through the more sensitive screens relating to social and emotional wellbeing. With inbuilt skip logic, the series of BCYR rubrics screen for mental health/SEWB, alcohol and other drugs tobacco use, and family and domestic violence as relevant to the individual woman. If a woman chooses images which reflect for example, tobacco use, the health professional may ask if the woman wants to address her tobacco use. If she does, a brief intervention or referral to “quit smoking” services or resources may be appropriate. If the woman is unconcerned about her tobacco use, usage is recorded. With regards to family and domestic violence, depending on answers to specific image selections made in explicit rubric questions, a safety planning tab may open. This enables the health professional to prompt the woman for more information and discuss with her what, if any, assistance or referrals she may want to put in place. The whole process culminates in a clinical events summary which sets out which self-directed supports the client requires and assists health professionals to record culturally safe ongoing care planning. Importantly, the woman’s “story” is a permanent entry in her health record, meaning that she is less likely to have to repeat her story in subsequent visits.

## Methods

### Study design

The Aboriginal Participatory Action Research method (APAR) was used in this qualitative study (32). Decolonizing approaches were also employed through Aboriginal research methodologies (32, 33), fostering knowledges beyond Western biomedical methods and critically examining relationships between colonization and specific maternal health outcomes (34). Yarning was chosen as the most appropriate Indigenous research methodology (49) applied as a decolonizing approach to encourage participants to share their stories through storytelling in line with cultural protocols (35, 36). As a rigorous research method, yarning is a preferred practice over semi structured interviews to facilitate authentic sharing of information between a researcher and Aboriginal participants (36, 49).

APAR confirms the primacy of Aboriginal women’s voices and worldviews, recognizing the need for researchers to work in partnership with the women to share their cultural knowledge in the reclamation of Aboriginal cultural knowledge systems. This approach also focuses on empowerment for Aboriginal communities and challenging power imbalances resulting from colonization (32).

### Setting

This study was undertaken with Aboriginal participants at a range of locations across the Perth metropolitan area and virtually with one regionally located participant.

### Governance and ethics

As part of the larger BCYR pilot study, this research was supported by the Aboriginal community within a project governance structure which included an Aboriginal Lead Research Advisory Group, an Elders Cultural Safety Group, and an Aboriginal Working Party Group to ensure co-design of the research, accountability, evidence gathering and analysis of findings. These groups were complemented and led by the Aboriginal Elders Advisory Council and Kaadininny Aboriginal Advisory Board at Ngangk Yira Institute for Change (19).

In consultation with these groups, the lead author, a Palawa woman and midwife, understood the cultural strengths of Aboriginal mothers and the need to address the risk deficit narrative and cultural biases commonly found in healthcare (3, 29). The Elders and Aboriginal Lead Research Advisory Group offered consistent advice and cultural governance to the research.

Ethical approvals were granted by (three committees, to be published on manuscript acceptance).

### Positionality statement

Two of the five authors are Aboriginal people. All authors are research academics with longtime connections with Aboriginal communities in urban, rural and remote areas of Western Australia.

### Inclusion criteria

Participants needed to identify as an Aboriginal person living in Western Australia, be engaged antenatally with a midwifery group, be aged over 16 years and able to converse in English.

### Recruitment

Convenience sampling using existing community networks were used to recruit eight Aboriginal women who were either pregnant or had a child within the previous 2 years. There are limitations with using convenience sampling, including motivation biases (37) and it is acknowledged the women who participated in this study were highly motivated. Strategies used to overcome the bias limitations and elicit high quality, thick data were culturally appropriate and sensitive methods, such as completing yarning in a location of the woman’s choice and building on participant engagement through deep listening (a highly valued Aboriginal method) while promoting credible and trustworthy dialogue (38) using the yarning method. Small population cohorts in qualitative research enable researchers to explore in-depth participant experiences that are embedded in unique social and cultural contexts. These types of investigations are not achievable in larger samples and are able to generate rich, thick data (39).

Aboriginal women who utilized the BCYR digital platform during their perinatal care at one of the pilot study sites were given the option by their health provider to participate in this research and were provided with an information sheet describing the research. After giving verbal consent, the women were contacted by the lead researcher to arrange a suitable time and location to conduct a yarning session. Prior to commencing the yarn, participants signed a consent form.

### Data collection

#### Quantitative data collection

Quantitative demographic data relating to the number of participants, their age, parity and identification as Aboriginal and Torres Strait Islander were collected.

#### Qualitative data collection

The lead author conducted all the yarns to ensure culturally safe and responsive participation, such as offering participants their choice of location for the yarn and using Dadirri, an Aboriginal concept of inner, deep listening which facilitates trusting, sensitive engagement and participation (40) and reassuring participants of confidentiality. This approach originated from the Ngangiwumirr culture in the Daly River region in the Australian Northern Territory and promoted by Dr. Miriam-Rose Ungunmerr-Baumann through the Miriam Rose Foundation (41).

The duration of the yarns was between 30 and 45 min, guided by open ended questions. All yarns were audio-recorded, transcribed verbatim, de-identified and returned to each woman for member checking. All participants authorized inclusion of their de-identified information in the analyses and reporting.

Participant responses are at risk of social desirability bias which can compromise the rigor of research findings and not adequately express participants’ feelings on the topic under investigation. The settings in which the data collection is undertaken and hierarchical positions between the researcher and participant can facilitate these biases. Conducive physical and psychosocial environments and sensitive interviewing are key to reducing these data errors (42). Yarning reduces potential bias issues by framing a culturally acceptable platform for information exchange.

### Data analysis

Collected demographic information was used to describe the cohort. Following qualitative data collection, the lead researcher undertook thematic analysis using a six-step approach to identify meanings and themes from the data, then discussed with and confirmed by two senior members of the research team in group analysis sessions (43). Emergent themes were further reviewed and confirmed by three Aboriginal health clinicians and a non-Aboriginal clinical psychologist. This systematic approach enabled consensus descriptions of the themes with robust Aboriginal lenses informing the analysis. Thematic coding was aligned with the seven domains of SEWB outlined in the conceptual model (Figure 1), illustrating how individual, family and community strengths and SEWB are influenced by connections to body, mind and emotions, family and kinship, community, culture, Country and spirituality. It is recognized that these are further influenced by social, cultural, political and historical determinants (2).

The small research population is noted with potential limitations for data saturation. However, data saturation can be attained within a small number of interviews, particularly with homogenous cohorts with distinct, narrowly defined research objectives (50, p. 9).

Member checks were facilitated through reflective debriefings on completion of the interviews. This was deemed necessary as repeat follow-up communication was not always achievable due to participants other responsibilities in their everyday lives (44, 45). The lead researcher also addressed potential biases through an audit trail which documented consistency of the research method across interviews, field notes, raw data and the stages of the six-step approach to thematic analysis (45).

## Results

### Demographic data

There were eight (*n* = 8) participants aged between 22 and 44 years of age, with four (*n* = 4) being first time mothers and four (*n* = 4) having more than one child. One woman identified as Aboriginal and Torres Strait Islander while the other women identified as Aboriginal from differing clan groups. There was one regional participant with the yarn conducted by phone; all other participants were in the metropolitan area. One interview was held in a woman’s home and six were completed at BCYR pilot sites.

### Qualitative data

Data were analyzed and interpreted through the decolonizing approach articulated by the SEWB framework (2). All these domains were evident across the women’s yarns about their experiences of using BCYR.

Six themes emerged from the data, one of which comprised a subtheme, reflecting Aboriginal mothers’ strengths during their perinatal journeys.

**Table 1:** 

Themes
Theme 1: Strengthening Identity-Reconnecting to Culture
Theme 2: Connection to Kinship/family Sub-theme: 2.1 Strong partner support
Theme 3: Connection to Country
Theme 4: Connection to Culture
Theme 5: Resilience and Self-efficacy
Theme 6: Women’s Experiences using BCYR

#### Strengthening identity-reconnecting to culture

A common theme for most participants was their sense of identity, with each disclosing either their strong connection to, or personal struggles with, their identity as Aboriginal women.

*“My cultural identity I’m carrying on my line. I’m carrying my grandmother’s line. I’m carrying on my parents’ line, so culturally that gives me a strength. I get to grow up my child and teach them what I did as a kid and what our culture is and where we come from and where our Country is, because that stuff that I all know and that’s something that like culturally helped me throughout my pregnancy”. (Participant 3)*

*“I had to prove myself too* [to Department of Child Protection-DCP]*, you know, that I’m a good mum. And then it gets me concerned because of little things like what I might do, like taking a six-week-old out bush, might not be ok for DCP…but that’s our identity. I feel like we’re looked down on, but our values, I mean we are family orientated people. Aboriginal people they are very family orientated people”. (Participant 1)*

Most participants identified that using the BCYR digital platform assisted in cementing a sense of cultural identity.

*“Preparing for baby. It’s such a new experience, and I’ve lived, you know, just looking after myself. So it was kind of that next stage of how I will be as a mum but also as like a partner and as a sister and how that will change my identity a little bit…so I think it’s just the fear of the unknown… but I’ve identified that* [through BCYR] *and I’m kind of just looking at other avenues to manage it…I know that I’ve had that for my whole life. I’m just being very, very overthinker…but it did get me thinking around. You know, it* [BCYR] *actually showed all the good stuff I have and all the supports I actually do have”. (Participant 6)*

*“My culture, and then all of a sudden I’ve got you know, cause too much family rivalry and stuff like that going on and I don’t wanna get involved, so I’ve taken a step back…and you know, then all of a sudden this* [BCYR] *app comes along with, you know, my culture on it… it was awesome. And then I was like wow…and when I saw that* [BCYR strengths] *tree, I was like, that’s basically it* [images reflecting my culture] *right there”. (Participant 5)*

#### Connection to kinship and family

Participants referred to the cultural practices and factors that positively contributed to their perinatal mental health. Most of the women discussed how kinship and family support was the most important strength relating to their wellbeing, giving them reassurance and a strong sense of connection to their culture. Just being listened to and having a sense of being cared for by family provided “massive relief, massive release, burden gone,” highlighted through use of BCYR. For example, Participant 4 responded to a question about what kept her strong during her pregnancy, saying *“Just having a lot of support from my family and partner. Especially, when it’s the last couple of weeks.”* Other participant’s examples which emphasized kinship/family connections were:

*“It’s family. So, it’s always been mum, my sisters and my support network…I mean my brothers are great too. Yeah, but it’s just different with sisters…with mum, when I need her. She is there…I mean our support system from our grandmother to my mum’s sisters is crazy as well”. (Participant 1)*

*“…I have my mum, I have my mother-in-law, I have all my aunties, I have this person, that person…It had me spinning off like all these strengths that I already had, so when it came down to BCYR, I pretty much had that in my head anyway…Friends of friends, cousins like you know, in laws they don’t have the same support as I did, and it made me have it like such an appreciation for it…Because you’re bringing it kind of into the light and BCYR brings your strength into the light. Yeah, it makes you appreciate”. (Participant 3)*

##### Strong partner support

While not all women had strong partner support, some participants described the importance of their partner’s care and support for both their physical and mental wellbeing.

*“I’m in really good state. I think personally I’m confident and I’m in happy place…100% my husband now is just beautiful…just so supportive”. (Participant 1)*

*“He* [partner] *doesn’t like seeing me in pain or stress. I’m just trying to prepare him that it is all normal. I need you to be the tough one…Definitely, I have a good one* [referring to her partner]. *We usually go out and do stuff together so I’m not trapped indoors by myself all the time. As it does get depressing. So I am pretty lucky there”. (Participant 7)*

#### Connection to country

Connection to Country emerged as an important theme with the majority of women identifying this as deeply strengthening their SEWB. It provides a place of grounding and belonging that also strengthens the women’s Aboriginal identity.

*“Going home bush, every Easter we go home and everyone comes. I’m talking like my nanna’s brothers and sisters…all their kids…and I mean I think there is like ten of them in the bush and everything. So, we all get together. Go home* [to Country]*. Just doing our normal sitting around the fire yarning, listening to the oldies. Just do that…it’s only once a year, but it gives you that strength to move through the year and then you look forward to it again next year… Just connected…home…safe. Yeah, just grounded. It grounds you again. Yeah, you get so busy with life, you go home, it’s only for four or five days we go out there and you come back and you’re grounded and you’re ready to move on with the year. And do it all again next year”. (Participant 1)*

*“Just the act of going out back out on country is what keeps me strong and my family so not necessarily just my immediate family, but like my, you know, going and seeing my cousins, my aunties and my uncles”. (Participant 3)*

Another woman described the need to go on Country when not feeling strong. It was a strategy to reconnect with herself and realign with her SEWB.

*“So every time that I don’t feel OK, every time that I feel stressed out, I pretty much will go back to somewhere that is a part of my Country. That’s the kind of things that I did or that I do when I’m not feeling strong and I can’t get that from the city because you need that as a blackfella, you need to go back out to where you are from to get that peacefulness and kind of like contentment…when you go back out* [to Country] *kind of like escaping that reality and getting a break and getting back your peace and then coming back and resetting…it just really clears the brain. Like it’s good mentally, like, mentally. Like when you feel full it’s like an outlet. It just resets everything”. (Participant 3)*

Several yarns from the women confirmed that many non-Aboriginal people have very limited understanding of the deep importance of connection with Country for Aboriginal women in pregnancy.

#### Connection to culture

Cultural factors contributing to the strengths of Aboriginal women’s perinatal mental health and wellbeing were highlighted, particularly their spiritual, ancestral and kinship connections as well as cultural practices such as smoking ceremonies, yarning, dancing and connecting with Country.

*“I do some traditional dancing with my cousins and my sister, and we use smoking as a way of just preparing, connecting with our Country, but also our ancestors as well. So I always use that as a big cleansing tool, especially, even with my baby shower. I had a smoking ceremony just to welcome, you know, baby into the world, even though she is still coming. And it was a good opportunity to share with non-Aboriginal friends as well. I feel like it shakes all negativity around. You know, if I have any doubts. If I’m feeling heavy, almost like if I’ve just, I feel like once I have a smoking, I’m cleansed, I feel lighter and I feel more connected. That’s how I personally take it”. (Participant 6)*

A few mothers raised the importance of passing on cultural knowledge and practices through kinship and Elders to the younger generations to maintain the strength and resilience of Aboriginal identity.

*“There’re people that are older than me, way older than me. That could be my parents and my people that are my own age, who have no connection to their Country, who when I say to them “Oh yeah, I go back out on Country and I go hunting” and that they’re like, “oh, we’ve never been hunting”. And I get sad because…that’s such an outlet for me…that’s just lost cause of the Stolen Generations and things like that…I’m privileged because I get to still do that, I still had a grandmother who was alive who could teach me that”. (Participant 3)*

#### Resilience and self-efficacy

Participants confirmed how Aboriginal women and their families continuously strive to overcome adversity through unique protective factors including connection to Country, connection to culture, family/kin, reclaiming language and cultural practices to enhance their self-efficacy and resilience. Acknowledging and drawing on these strengths during the perinatal period was evident throughout all yarns with the women.

One woman stressed the importance of family and kin to strengthen her during her pregnancy and postnatal period, enhancing her self-efficacy and sense of control.

*“Strength of my family, like the closeness and support which like…I don’t want to sound racist. A lot of Wadjela* [non-Aboriginal] *families don’t have that. They don’t have that closeness. Don’t have that value of family, whereas in black fella families like you have that value and that’s what kept me strong, especially when I came home…that’s one thing that kept me pretty at peace when I was pregnant, too. Having a good support system around me, people that cared and love me…I don’t invalidate my feelings, but to a degree I kind of do because someone is worse off…worse off than me. But at the same time I need to do what I need to do to get better, but don’t go into a well of poor me, poor me because there’s actually some women out there* [who are worse off]”*. (Participant 3)*

A second woman recognized the ability of BCYR to identify and help her to address her concerns which highlighted her self-efficacy and empowerment.

*“Yeah, absolutely…I was identifying that and realizing, you know, that that’s a little bit of a worry but also…Yeah, kind of thinking…unpacking it a little bit more and saying like…why is that a worry or what can I do to kind of manage it as well. Yeah.* [BCYR] *gives you a bit more power to say like, OK, this is it. This is what I wanna deal with. Let’s go for it, you know. 100% and like unpack it a little bit more…I spoke about like my mum and like why and I was like OK there was a little bit of, you know, head butting and just it’s not, wasn’t always harmonious, but it was like oh it kind of brought some of those emotions back up a little bit”. (Participant 6)*

A further participant described how using BCYR enabled her to recognize and yarn about mental health concerns with her health practitioner, valuing the opportunity to look after their own mental health and enhancing a sense of self-efficacy, control and resilience during her pregnancy.

*“Because for me, I think that the more we talk about mental health and that kind of stuff, it gets talked about more through other agencies and stuff like that. And then we can actually do more. It shouldn’t be a taboo topic because a lot of us deal with it. Yeah, and I’m a huge advocate for mental health. I think the more we talk about it, the more that can be done…with this one* [current pregnancy] *it was like, oh, we’ve got a psychologist, and we can put you on to this one and this one. And it was like, wow”. (Participant 5)*

#### Women’s experiences using BCYR

Women’s experiences of using BCYR were explored, highlighting their strength-based perspectives of using the platform. Several women identified that the flow of images and Aboriginal voice overs reinforced their own strengths and resilience, as well as encouraging them to be self-determining in addressing any worries throughout their perinatal care. All women were able to use the BCYR app with no additional training.

*“I’ve identified all the positive influence, or factors and resources that I do already have. So, as I said*…[BCYR] *was a great reflection piece for me… BCYR brings your strength into the light. Yeah, it makes up an appreciation. But it also like I don’t know, keeps you real”. (Participant 3)*

*“BCYR* [is about] *how you’re feeling, but also your strengths, your weaknesses, things that you can work on, things that you have already got going for you…BCYR doesn’t make you feel so pressured like there might be something wrong”. (Participant 1)*

*“*[BCYR] *made me stop and make me realise as strong as I am, I still have weak spots, and the support that I have around me”. (Participant 2)*

*“*[BCYR] *was pretty straightforward and pretty easy to follow along…especially being Aboriginal, seeing all those pictures and questions that made things easier to understand”. (Participant 4)*

Participants also reflected on health professionals’ engagement with their clients through the BCYR platform.

*“I think she [midwife] got to know me a bit better. On a different level. Personal, because there was some questions on there you that you know you wouldn’t feel comfortable talking to them and instead you could just show them, you know…* [and the midwife] *is more understanding”. (Participant 8)*

*“Before we did this* [BCYR], *she didn’t really know at me at all…think she [child health nurse] understands me more on a personal level”. (Participant 2)*

*“…you’re basically seen as a vessel for this baby…but they [midwives] forget about you…Where this* [BCYR] *didn’t, you know it checked on me and I was telling her [midwife] about my sciatica and my mental health and stuff like that…So having that* [BCYR and relationship] *with her is what made me feel a lot more connected. But I’ve said I’ve never experienced anything like this and I wish that I could have more of it”. (Participant 5)*

## Discussion

The aim of this study was to explore Aboriginal women’s resilience, self-efficacy and empowerment during their perinatal experiences, assessing factors contributing to their cultural strengths when addressing perinatal mental health concerns. As such, there was a focus on understanding Aboriginal women’s perspectives not only of their mental health but also what helps them maintain a strong sense of self during a time in their lives when exposure to health care can render them vulnerable to the external pressures and expectations of a system that does not meet their needs. The study outcomes demonstrate how Aboriginal women were able to identify their strengths in the perinatal period when experiencing a co-designed and culturally responsive process, and how this helped them understand and appreciate their strengths as grounded in their connections to family and culture which in turn assisted them to acknowledge and address their perinatal concerns.

Recent research exploring culturally safe Aboriginal-led strength-based best practice approaches to addressing the complex and multilayered issues which impact Aboriginal people reports that emerging national and international evidence demonstrate positive impacts of self-determination and cultural continuity. These cultural elements provide psychosocial protection against systemic racism, in addition to empowering the cultural determinants of wellbeing (5, 29).

There was an interplay of concepts across the six themes with most women identifying their key cultural strengths as situated in connections to culture, Country and family/kin, all of which are reflected in the SEWB framework (2). Gee and colleagues note that while the SEWB of the self is connected to domains encompassing spirit, spirituality and ancestors, body and behaviors, mind and emotions, family and kinship, community, cultural and Country and land; these domains are interconnected and impacted by cultural, social, historical and political determinants. They further highlight that expressions and experiences of SEWB over the life span will ebb and flow as risks disrupt connections, while protective factors restore and strengthen connections. The authors note that resilient and empowered individuals and communities have the capabilities to take full advantage of protective factors while taking steps to avoid circumstances of risk (2). In our analysis, it was evident that potential risks were offset by the strengths that women perceived came from their ability to step outside day-to-day life and step into cultural practice – using visits to Country, smoking ceremonies and support from family to navigate their perinatal journeys. Aboriginal women were surprised and impressed by their experiences of using the BCYR digital platform and expressed that their connections to health professionals (midwives and child health nurses) were positively improved. The women felt empowered to discuss their concerns and self-direct how they would address these.

The analysis identified the factors contributing to perinatal Aboriginal women’s resilience when using a strength-based approach to assessing their social and emotional wellbeing. The potential of BCYR to support women in recognizing their protective factors which enhance their ability to negotiate their own culturally safe perinatal mental health pathways has been identified and examined. This has strong potential to enrich their empowerment to articulate impacts of racism and to navigate their own unique pathways toward psychosocial and cultural wellbeing support (5, 6, 23).

Participant comments confirmed that the process of reflecting on these cultural elements through the BCYR app were empowering, enabling them to recognize their perinatal mental health strengths and protective factors through culturally safe yarning with their midwife or child health nurse, exploring their Aboriginal identity and self-identification of confidence in their self-efficacy and resilience (2, 3, 29). With the current EPDS perinatal screening tool being increasingly recognized as having limitations in Aboriginal contexts, particularly in the area of Aboriginal women’s self-efficacy, strengths and resilience (12, 14, 16, 17), these study participants’ strong engagement with the BCYR program in addition to their willingness to discuss sensitive issues and identify their cultural strengths confirms the value of the program as an empowering, acceptable, strength-based psychosocial assessment tool able to facilitate elements of self-efficacy and resilience. This endorses a crucial recommendation for health service policy and clinical strategies to recognize the EPDS as a challenging and alienating process for Aboriginal women with embedded cultural biases which fail to recognize the cultural elements described in this study (12, 16, 17, 21).

In other studies, Aboriginal mothers have noted that some clinicians were unable to provide appropriate support, acknowledgement or follow up care for their mental health concerns when these were raised when using the EPDS (3, 12, 14, 16, 17, 30). They also stated that midwives and child health nurses did not fully understand their personal lives, thereby highlighting racial-empathy gaps which enhance disparities within standard models of care. In contrast, using the innovative co-designed BCYR program in clinical practice, which aims to improve maternal and infant health and wellbeing outcomes, seems to empower mothers to have authentic control over their perinatal assessment and follow-up care. Additionally, BCYR can provide clinicians with deeper, more contextual understandings of mothers’ concerns through shared navigation of BCYR prompts, images and voice-overs and use of deep, non-judgemental listening. The screening process which BCYR promotes guides mothers and clinicians toward improved culturally safe perinatal resources and facilitates strategies for clinicians to develop stronger partnership approaches with mothers when exploring protective factors and strengths (12, 23).

Participants confirmed their use of BCYR was culturally safe and respectful of their past and current experiences with the strength-based images significantly supporting their self-efficacy and resilience; with strong potential to enhance Aboriginal mothers SEWB (12, 46). Importantly, this study has confirmed the value of a decolonizing lens to explore how the mothers’ protective factors and strengths can mitigate their perinatal mental health challenges, not evident in Western understandings of resilience. However, it is recognized that the concept of resilience will vary across diverse Aboriginal populations nationally, with recommendations for local research with individual communities to facilitate their authentic contextual understandings of what drives their resilience (22).

As such, findings from this study demonstrate the applicability of BCYR as a contemporary, empowering, and clinically relevant means of undertaking crucial perinatal mental health and other health behavior assessments. Use of the BCYR digital platform may assist in addressing inequities in delivery of maternal health care, providing flexibility for health professionals to respond to Aboriginal women’s self-identified health care needs at a local level, and notably, highlighting women’s resilience as a factor for inclusion in culturally safe models of care. This has wide ranging implications for practice, with contemporary perinatal models of care needing to acknowledge the centrality of Aboriginal women in the identification of their own unique physical, psychosocial and cultural strengths and challenges impacting on their health and wellbeing (12). Further, working in authentic partnerships facilitates health professionals enhanced understanding of acceptable and accessible culturally safe perinatal resources and strategies relevant to the Aboriginal communities they work with (19, 21). These approaches also highlight the essential requirement for practitioners, along with government and non-government health and social service agencies to develop genuine strength-based approaches within antenatal clinical practice and policies. Recognizing cultural identity affirms the value of these approaches in enhancing SEWB outcomes during this critical period for mothers and their children (2, 3, 12, 29, 46).

This research has also identified areas for further research. It is recommended that investigation is undertaken to identify and critically examine further opportunities for Aboriginal women to incorporate their connection to culture and identity, family and Country and cultural practices to build and strengthen their identity, clinical pathways and resilience through the perinatal period. In partnership with Aboriginal women, it is also proposed that clinical practice and policies are explored to ascertain how findings from this current research can be embedded into perinatal health care practice, and strategies for promoting strength-based culturally safe care and the women’s SEWB (19).

## Limitations

This research was undertaken in urban communities located in Perth, Western Australia, and one rural Western Australian location. There are unique geographical, cultural and social differences and challenges unlike other national and international localities. Nonetheless, the research methods used in this study resulted in outcomes which contribute to the outcomes of the larger study, which when combined, critically inform ongoing development of culturally safe perinatal support for Aboriginal women in a range of settings.

There is potential for bias in participant selection through convenience sampling. It is recognized that highly motivated women chose to be included in the study. However, there was no indication of disinterest in the BCYR program by women who chose not to participate, rather not having enough time to be included or having competing interests such as family commitments.

The possibility of convenience sampling leading to selection bias has been acknowledged and described earlier. While women self-selected their involvement in this research due to their participation in the BCYR pilot implementation, research participants were representative of the broader population of BCYR participants.

## Recommendations

There is potential for these research outcomes to inform a partnerships approach to culturally safe policy and practice in relation to other perinatal populations both nationally and internationally. However, further investigations would need to be undertaken to explore essential elements such as reliability of digital access, participant engagement, program sustainability and workforce reliability and training.

## Conclusion

This research has highlighted Aboriginal women’s strengths, identity, resilience and self-efficacy in the perinatal period established through their use of the BCYR digital platform. The importance and effectiveness of using the platform to build women’s relationships with health professionals has been confirmed, as has the benefit of identifying and valuing women’s cultural strengths. In turn, this enhances Aboriginal women’s empowerment to access culturally safe support to mitigate their perinatal mental health challenges and augments the cultural competence of health professionals and the broader health system.

## Data Availability

The raw data supporting the conclusions of this article will be made available by the authors, without undue reservation.
